# Expert consensus for pertussis in children: new concepts in diagnosis and treatment

**DOI:** 10.1007/s12519-024-00848-5

**Published:** 2024-11-14

**Authors:** Yu-Mei Mi, Ji-Kui Deng, Ting Zhang, Qing Cao, Chuan-Qing Wang, Sheng Ye, Ying-Hu Chen, Han-Qing He, Bei-Bei Wu, Yan Liu, Mei Zeng, Wei Li, Fang Wu, Hong-Mei Xu, Shi-Yong Zhao, Gang Liu, Wang Hua, Dan Xu, Guan-Nan Bai, Ying Yang, Li-Su Huang, Yi-Ping Chen, Kai-Hu Yao, Zhu-Jun Shao, Chun-Zhen Hua

**Affiliations:** 1 Department of Infectious Diseases, Children′s Hospital, Zhejiang University School of Medicine, National Clinical Research Center for Child Health, Hangzhou, 310052 China; 2 Department of Infectious Diseases, Shenzhen Children′s Hospital, Shenzhen, 518038 China; 3 Department of Infectious Diseases, School of Medicine, Shanghai Children′s Hospital, Shanghai Jiao Tong University, Shanghai, 200262 China; 4 Department of Infectious Diseases, Shanghai Children′s Medical Center, Shanghai Jiao Tong University School of Medicine, Shanghai, 200127 China; 5 Department of Nosocomial Infection Control, Department of Clinical Laboratory, Children′s Hospital of Fudan University, Shanghai, 201102 China; 6 Department of General Intensive Care Medicine, Children′s Hospital, Zhejiang University School of Medicine, National Clinical Research Center for Child Health, Hangzhou, 310052 China; 7 Department of Immunization Program, Zhejiang Provincial Center for Disease Control and Prevention, Hangzhou, 310052 China; 8 Department of Microbiology, Zhejiang Provincial Center for Disease Control and Prevention, Hangzhou, 310052 China; 9 Department of Immunization Program, Hangzhou Center for Disease Control and Prevention, Hangzhou, China; 10 Department of Infectious Diseases, Children′s Hospital of Fudan University, Shanghai, 201102 China; 11 Department of Clinical Laboratory, Children′s Hospital, Zhejiang University School of Medicine, National Clinical Research Center for Child Health, Hangzhou, 310052 Zhejiang China; 12 Department of Chinese Medicine, Children′s Hospital, Zhejiang University School of Medicine, National Clinical Research Center for Child Health, Hangzhou, 310052 China; 13 Department of Infectious Diseases, Chongqing Children′s Hospital, Chongqing, 400015 China; 14 Department of Infectious Diseases, Hangzhou Children′s Hospital, Hangzhou, 310014 China; 15 Department of Infectious Diseases, Beijing Pediatric Research Institute, National Center for Children′s Health, Beijing Children′s Hospital, Capital Medical University, Beijing, 100045 China; 16 Department of Infectious Diseases, Yuying Children′s Hospital of Wenzhou Medical College, Wenzhou, 325003 China; 17 Key Laboratory of Major Diseases in Children, Ministry of Education, National Clinical Research Center for Respiratory Diseases, National Key Discipline of Pediatrics, Laboratory of Infection and Microbiology, Beijing Pediatric Research Institute, National Center for Children’s Health, Beijing Children′s Hospital, Capital Medical University, NO.56 Nanlishi Road, Xicheng District, Beijing, 100045 China; 18 National Key Laboratory of Intelligent Tracking and Forecasting for Infectious Diseases, Chinese Center for Disease Control and Prevention, National Institute for Communicable Disease Control and Prevention, Changping, P.O.Box 5, Beijing, 102206 China

**Keywords:** Children, Diagnosis, Expert consensus, Pertussis, Treatment

## Abstract

**Background:**

Pertussis resurgence has been reported worldwide in the past two decades. Pertussis is still endemic and difficult to control though with universal vaccination in children. The resurgence may be related to multiple variables, such as increased disease awareness and laboratory tests, waning of immunity following vaccination, and/or genetic mutations of *Bordetella pertussis*. For better pertussis prevention, diagnosis, and management, we called up an expert panel to develop this expert consensus to provide new concepts in diagnosis and treatment for clinical practice.

**Data sources:**

The expert groups collected clinical evidence, summarized their clinical experiences, evaluated preliminary recommendations or guidelines, and then organized open-ended discussions to form the recommendations. This consensus was developed by reviewing the literature and studies in databases, including PubMed, Cochrane, EMBASE, the China Biomedical Database, and the Chinese Journal Full-text Database up to May 2024. The search terms included “pertussis” or “whooping cough”, “children”, “diagnosis”, and “treatment”.

**Results:**

The burden of pertussis has also changed from infants to  school children and adults, and these age groups have consequently become the main source of infection for vulnerable population including infants and newborns. In China, a high prevalence of erythromycin-resistant *Bordetella pertussis* (ERBP) has been reported in the past decade. ERBP may lead to failed clinical empirical treatment with macrolides, which poses a great challenge for pertussis management and control. For better management of pertussis, a flow diagram for diagnosis and treatment of pertussis was presented in this consensus. This consensus also described the diagnostic criteria for pertussis, high-risk cases, and severe pertussis. Macrolides can still be used to treat confirmed erythromycin-sensitive *B. pertussis* (ESBP) infections, whereas oral trimethoprim–sulfamethoxazole therapy is the initial treatment option for children older than two months. For infants younger than two months, severe patients, or those exhibiting a high degree of sulfonamide allergy, intravenous administration of piperacillin or cefoperazone–sulbactam is advised.

**Conclusions:**

This expert consensus provides a comprehensive guidance and a reference for the diagnosis and treatment of pertussis in children.

**Graphic abstract:**

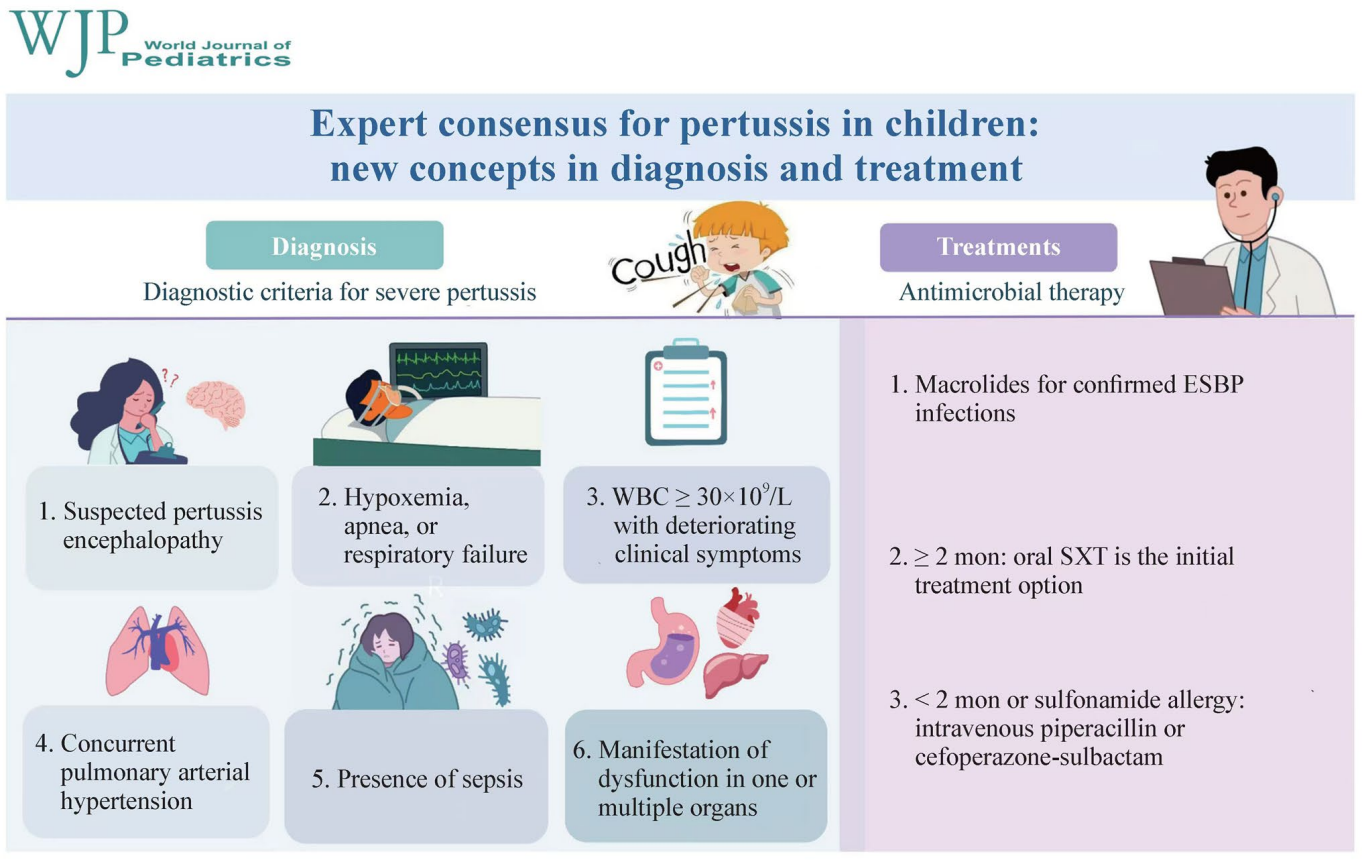

## Introduction

Pertussis, also known as whooping cough, is a highly transmissible respiratory disease caused by *Bordetella pertussis* (*B. pertussis*) [1]. Despite high vaccination rates in children, pertussis resurgence has been reported in many developed countries due to immune attenuation and B. pertussis mutation after widespread vaccination [2–4]. The incidence of pertussis decreased and was once well controlled in China after the pertussis vaccine was included in the Expanded Program on Immunization (EPI) in 1978. However, over the past decade, the incidence of pertussis worldwide has demonstrated a consistent upward trend, with a yearly increase. This increasing trend, particularly from 2017 to 2019, revealed that the number of reported cases surpassed 30,000 in 2019, echoing infection levels reported in the late 1980s [5]. Since the COVID-19 epidemic in December 2019, seasonal temporal trends of pertussis have changed from a peak in August to an erratic growth or decline of cases throughout the years, with an unusual rise in winter. The number of cases rose gradually from 538 in February 2023 to 1512 by June and then doubled monthly, reaching 9126 in December. This trend persisted, with the number of cases increasing to 408,712 between January and July in 2024 [6]. The burden of pertussis has shifted from infants to older children and adults, and these age groups have consequently become the main source of infection for infants [7]. Cases of pertussis in preschool- and school-aged children were found increased, and severe patients were commonly found in newborns and infants. Furthermore, erythromycin-resistant *B. pertussis* (ERBP) has become prevalent, since the first resistant isolate was identified in 2012 [8]. While ERBP has long been identified in the United States and Europe [9, 10], its prevalence has not been detected in these regions. Notably, ERBP accounted for 48.6–100% of the clinical *B. pertussis* isolates investigated from 2013 to 2022 in China, and the minimum inhibitory concentration (MIC) values of ERBP strains often surpass the highest measurable thresholds (> 256 mg/L) [7, 11, 12]. The prevalence of ERBP differs from the epidemiology of normal pertussis in other countries, which creates some challenges for clinical management. To further standardize the clinical diagnosis and treatment of pertussis, the expert group collected clinical evidence, summarized clinical experience, evaluated preliminary recommendations or guidelines, and then organized open-ended discussions to form the recommendations. We called up an expert panel, including specialists from pediatric infectious diseases, microbiology, infection and immunology, clinical laboratory medicine, disease prevention and control fields, and Chinese medicine. This consensus was developed by reviewing the literature and studies in databases, including PubMed, Cochrane, EMBASE, the China Biomedical Database, and the Chinese Journal Full-text Database up to May 2024. The search terms included “pertussis” or “whooping cough”, “children”, “diagnosis”, and “treatment”. It has been registered on the website of the Practice Guideline REgistration for TransPAREncy (PREPARE, website: http://www.guidelines-registry.cn/), and the registration number is PREPARE-2024CN557.

## Etiology

*B. pertussis* belongs to the *Bordetella * genus in the family *Alcaligenaceae*. It is a gram-negative, rod-shaped, obligately aerobic, nonflagellated bacterium. *B. pertussis* produces a wide array of virulence factors, including numerous toxins and biologically active products that play crucial roles in its pathogenesis and immunity. The most important virulence factor is pertussis toxin (PT), which has promitogenic activity, affects lymphocyte circulation, and acts as an adhesive agent through which bacteria bind to respiratory ciliated cells. *B. pertussis* is weakly resistant to physical and chemical factors [13]. It can be inactivated at 56 °C for 30 min, after exposed to sunlight for 1 h, or dried for 3–5 h. It is sensitive to ultraviolet rays and general disinfectants.

## Clinical presentations

The typical disease progresses through three distinct clinical stages: the catarrhal phase (1–2 weeks), the paroxysmal phase (2–6 weeks), and the convalescent phase. The overall duration of the illness typically ranges from about 6–12 weeks, with some cases lasting longer. Fever is usually absent, and if it is present, it is generally fleeting and mild and occurs during the catarrhal phase, which is highly contagious. During the paroxysmal phase, individuals typically experience prolonged, repeated, or violent coughing episodes characterized by a high-pitched "whoop" sound as they finally inhale at the end of a coughing fit. Relief from a cough is often achieved only after the patient expels viscous sputum. Paroxysmal cough occurs frequently at night, disturbing sleep, and even causing insomnia in older children and adolescents. However, in patients without mixed infections, a few significant abnormalities are observed during the non-cough episodes. As the illness progresses, vomiting or retching is often observed at the end of a coughing episode. Patients who experience frequent coughing episodes may develop tongue frenulum ulcers, facial and eyelid edema, sub-conjunctival hemorrhage, epistaxis, and, in severe cases, intracranial bleeding. Newborns and infants under 6 months of age with pertussis often exhibit paroxysmal cyanosis, even severe presentations including apnea, convulsions, bradycardia, or even cardiac arrest.

During the convalescent phase, the paroxysmal cough gradually diminishes in intensity and frequency, with the distinctive "whoop" sound fading. Nevertheless, it is noteworthy that children may relapse into paroxysmal coughing episodes if they encounter cold stimuli, secondary infections caused by other pathogens, passive smoking, or other detrimental factors during this recuperation phase.

Possibly due to vaccination and the maturation of organ functions with increased age, pertussis in older children, adolescents, and adults often presents with atypical symptoms, whose clinical staging is not obvious [5]. These patients may lack typical paroxysmal cough, inspiratory “whoop”, and vomiting at the end of cough. Some patients may experience a shortened illness duration, leading to a clinical process resembling a mild cold [5]. Additionally, more patients may exhibit persistent or chronic cough and are often neglected because of mild symptoms. Some patients are even considered asymptomatic *B. pertussis* infections. Other atypical symptoms include paroxysmal dyspnea at night and intermittent sweating. When patients have underlying diseases related to cough, such as asthma, or co-infection with other pathogens, typical pertussis symptoms are less likely to be identified.

## Complications

Pertussis can result in significant complications, particularly among infants younger than 6 months [1]. Pneumonia is the most prevalent complication and is often triggered by various bacteria, including *Moraxella catarrhalis*, *Streptococcus pneumoniae*, *Staphylococcus aureus*, and *Haemophilus influenzae*, as well as viruses such as *adenovirus* and *respiratory syncytial virus* [14]. Pneumonia triggered by the primary causative agent, *B. pertussis*, is uncommon. In some children, it may lead to the development of atelectasis, pneumothorax, mediastinal emphysema, subcutaneous emphysema, umbilical hernia, incarceration of inguinal hernia, and rectal prolapse. Infants are particularly vulnerable to asphyxia. Adolescent and adult patients may endure rib fractures and periorbital subcutaneous hemorrhages. In severe cases, pertussis encephalopathy, pulmonary hypertension, respiratory failure, bacteremia [15, 16], or sepsis [17, 18] can occur, primarily during the paroxysmal phase, potentially leading to sudden death [19].

## Laboratory examinations

### Blood routine examination

Peripheral blood white blood cell (WBC) count increased markedly, usually 20–50 × 10^9^/L, at the end of the catarrhal period and during the spasmodic paroxysmal coughing phase. Notably, lymphocytes tend to be the predominant cell type. Conversely, C-reaction protein (CRP) levels are generally within the normal range. Notably, WBC and lymphocyte counts can vary significantly among patients, depending on their duration of illness or age group. In vaccinated individuals or those with prior *B. pertussis* infection, WBC counts often remain within normal limits, which indicates that leukocytosis is not a sensitive indicator of pertussis [20, 21]. While specific studies have reported the presence of fissure lymphocytes in the peripheral blood of children with pertussis [22], this finding alone is not indicative of a diagnostic basis. Blood chemistry should be tested for monitoring liver and kidney function in hospitalized patients.

### Etiologic detection

#### Bacterial culture of *B. pertussis*

*B. pertussis* can be isolated from respiratory excretions, usually nasopharyngeal swabs, through culture techniques. A higher positive culture rate is observed among patients who are in the catarrhal stage of the infection or those who have not received treatment with sensitive antibiotics [21]. It is advisable to utilize calcium alginate swabs for sample collection, ensuring prompt inoculation of the collected samples. For primary culture, the recommended medium is selective carbon agar supplemented with cephalexin. The isolated strains can be used to assess antimicrobial susceptibility, providing crucial reference information for the selection of antimicrobial agents for clinical treatment. Although *B. pertussis* was isolated by culture from blood samples from pertussis patients in a previous study [15, 16], routine blood culture is not recommended for detecting *B. pertussis* due to the extreme rarity of *B. pertussis* bacteremia.

#### Nucleic acid detection for *B. pertussis*

The fluorescent quantitative polymerase chain reaction (qPCR) method is frequently used to detect nucleic acids in *B. pertussis* in clinical settings. The samples may include oropharyngeal or nasopharyngeal swabs, sputum, or alveolar lavage fluid. *B. pertussis* nucleic acid can be detected in respiratory specimens from pertussis patients. In recent years, next-generation sequencing (NGS) techniques have been widely used to detect unknown pathogens in patients with suspected infections. Surprisingly, nucleic acid from *B. pertussis* was found in the blood samples of patients with severe pertussis [17, 18]. These patients were defined as having bacteremia or bloodstream infection due to the severity of their conditions in these reports [17, 18]. However, this definition has not been consistently recognized and adequately verified.

### Serological tests

Serum pertussis-specific antibodies titers elevate are elevated in pertussis patients during the recovery period. Testing for IgG antibodies against pertussis toxin (PT) is advised for those with a duration exceeding 1 month. As the most widely used single serum assay, PT-IgG provides valuable insights for retrospective diagnosis. Nevertheless, its breakpoint value is still being determined. Serological diagnosis is most reliable in patients who have not received a recent vaccination, as it is based on a fourfold or greater increase in the antibody titer from acute to convalescent samples.

### High-risk patients who need *B. pertussis* detection

Early etiological diagnosis in children with pertussis can help initiate targeted antimicrobial therapy as soon as possible, reducing coughing symptoms, shortening the duration of the illness, and decreasing complications. Moreover, timely isolation of pertussis patients can significantly reduce secondary transmission. Medical institutions should strive to perform etiological detection techniques, such as polymerase chain reaction (PCR) testing, bacterial culture, or testing for IgG antibodies against pertussis toxin for *B. pertussis*. All children who meet any of the following conditions should be tested to confirm the presence of *B. pertussis* infection, regardless of whether they have asthma, allergies, or other specific cough histories or diagnoses.

1. Infants and young children (< 5 years of age) without a history of pertussis immunization who have been coughing for at least 1 week, as well as older children and adolescents who have been coughing for at least 2 weeks.
2. Having pertussis-like cough: A cough is characterized by one or more typical pertussis traits, including paroxysmal cough, "whooping" sounds, posttussive vomiting or coughing intensifies at night,” with no significant abnormalities observed between coughing episodes.
3. Infants experiencing recurrent episodes of apnea, choking, cyanosis, or bradycardia, unexplained by any other reasons.
4. Children who have had close contact [23] with pertussis patients and subsequently develop cough symptoms of any duration.
5. Children who are experiencing coughing without notable fever, reside in a household with multiple members suffering from chronic cough, or attend a school or daycare center where instances of pertussis occur.
6. Children with symptoms of cough who have significantly elevated WBC counts and/or lymphocyte counts.
7. Children without a prior history of asthma who have reported experiencing paroxysmal nocturnal respiratory distress following a cold.
8. Newborns or infants who have not received pertussis vaccination and who presented with severe pneumonia during the pertussis epidemic.
9. Infants with sudden death syndrome might have symptoms of cough; alternatively, their family members may have suspected pertussis.

## Imaging

### Chest radiography

The incidence of pertussis concurrent with pneumonia is notably high among young infants [21], and chest X-ray examination is a valuable tool for detecting lung lesions. Notably, in the absence of co-infection, individuals afflicted with pertussis often display a limited number of radiographic abnormalities that do not correlate with the severity of clinical manifestations, particularly in older children and adolescents with pertussis.

### Echocardiography

For infants, routine echocardiography and evaluation of pulmonary artery pressure are recommended. Additionally, children with pulmonary hypertension necessitate close monitoring.

### Establishing the diagnosis

A comprehensive analysis of the epidemiological background, clinical presentation, and laboratory test results is needed for the diagnosis of pertussis. Only through a meticulous and methodical approach can a definitive diagnosis be made. Even during seasons where other pathogenic infections are prevalent, pediatricians must remain vigilant and alert to the possibility of co-infection with *B. pertussis*. Maintaining a high level of suspicion and taking the necessary steps to ensure accurate diagnosis and timely treatment can help mitigate the risk of this potentially severe respiratory illness.

### Clinical diagnosis

The presence of any of the following clinical characteristics may indicate a clinical diagnosis of pertussis.

- (1) Suspected cases of pertussis with peripheral blood leukocyte counts of ≥ 20 × 10^9^/L and lymphocyte counts of ≥ 10 × 10^9^/L [21, 24];
- (2) Exhibiting typical clinical symptoms of pertussis and having a clear epidemiological link to confirmed cases of pertussis.

### Confirmatory diagnosis

Confirmatory diagnosis is frequently alluded to as a laboratory diagnosis. Patients present with either typical or atypical clinical manifestations of pertussis coupled with the presence of one or more of the following etiological evidence can be diagnosed:

1. Bacterial culture: *B. pertussis* was isolated from respiratory secretions, including nasopharyngeal oropharyngeal swabs, sputum, and bronchoalveolar lavage fluid.
2. Positive outcomes of nucleic acid detection of *B. pertussis* in respiratory secretions through techniques such as qPCR and NGS.
3. Serological testing: for those who have not been recently vaccinated with the pertussis vaccine, a notable increase of at least fourfold in the serum PT-IgG titer was detected during the recovery phase compared with the acute phase. Alternatively, individuals who have not received a pertussis vaccine in the past year or who never had whooping cough have a single dose of PT-IgG antibody titer above the recommended threshold for the diagnosis of acute infection, as stated in the product insert.

### Severe pertussis

Patients with severe pertussis suffer from a significantly elevated risk of mortality, underscoring the paramount importance of early identification and prompt intervention to optimize the success rate of treatment. The high-risk cases for severe pertussis in children include the following: (1) infants aged three months or younger [24, 25]; (2) absence of vaccination or incomplete primary immunization with pertussis-containing vaccines; (3) premature birth, newborns with low birth weight, or those with an Apgar score below 8 [25, 26]; (4) presence of underlying conditions, including severe congenital heart disease; (5) peripheral blood leukocyte count exceeding 20 × 10^9^/L and lymphocyte count exceeding 10 × 10^9^/L [24, 27]; (6) manifestation of apnea or cyanosis following the onset of symptoms; and (7) coexistence of mixed infection or concurrent pneumonia [25, 27].

There is currently no unified standard for diagnosing severe pertussis. To address this gap, we have made a comprehensive literature review on the characteristics of severe pertussis and integrated clinical experiences of the experts in the panel. Herein, we propose diagnostic criteria for identifying severe pertussis in children. Severe pertussis can be diagnosed if the patients meet any of the following criteria [28–31]:

1. Suspected pertussis encephalopathy manifesting as extreme fatigue, anorexia, and seizure attacks;
2. Frequent or persistent hypoxemia, apnea, or respiratory failure;
3. With an extremely high peripheral blood leukocyte count up to 30 × 10^9^/L which is increasing continuously, accompanied by deterioration of clinical symptoms;
4. Concurrent pulmonary arterial hypertension;
5. Presence of sepsis;
6. With one or multiple organ dysfunctions.

**Fig. 1:**
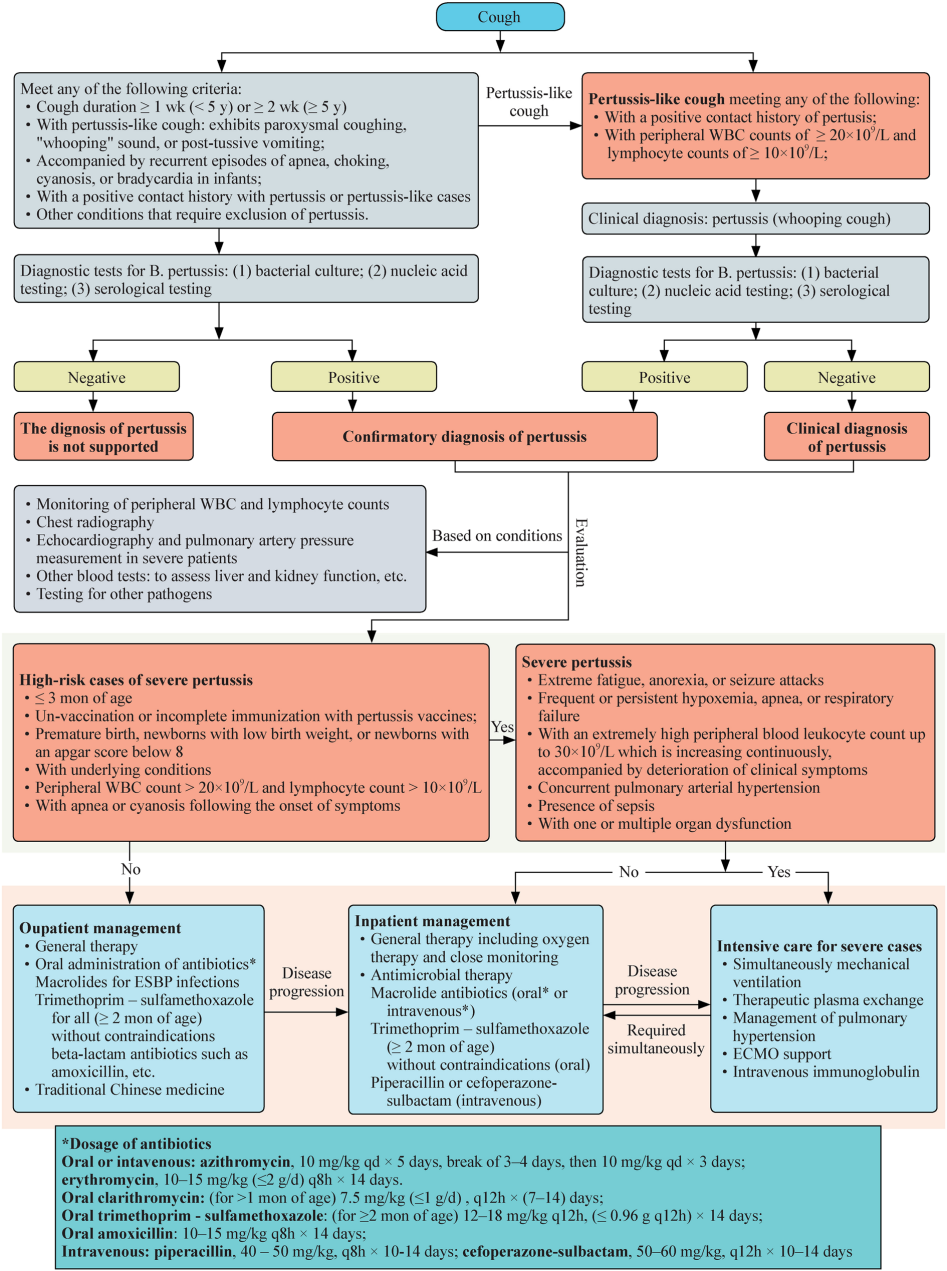
The diagnosis and treatment flow diagram for children with whooping cough

## Differential diagnosis

### Disease with pertussis-like cough caused by other pathogens

Children presenting with a paroxysmal cough similar to pertussis but testing negative for *B. pertussis* should be further assessed for potential infections caused by alternative pathogens. This includes but is not limited to *Bordetella parapertussis* [32], other *Bordetella* species, respiratory viruses, and *Mycoplasma pneumoniae*. Conducting appropriate testing for these pathogens can aid in accurate diagnosis and differentiation for specific pathogens. The most common mistake here is ruling out pertussis diagnosis solely on the basis of a negative test for etiology (bacterial culture and nucleic acid testing), which is negative in most parts of the pertussis course and could be negative occasionally because of the sampling technique and so on. In cases where a patient is diagnosed with "pertussis syndrome" or "pertussis-like syndrome", it is crucial to identify the specific pathogen responsible for the illness to distinguish it clearly from pertussis. The use of "pertussis syndrome" or "pertussis-like syndrome", which is no more rational than the use of a "cough of unknown origin", should not be considered as the final diagnosis.

### Tuberculous bronchial lymphadenitis

Tuberculous bronchial lymphadenitis manifests as enlarged mediastinal lymph nodes that exert pressure on the trachea and bronchi, resulting in a chronic spasmodic cough reminiscent of pertussis. However, individuals suffering from this condition usually have a prior history of tuberculosis exposure, accompanied by symptoms indicative of tuberculous intoxication. Moreover, the presence of primary tuberculosis lesions in the lungs and enlarged lymph nodes visible on pulmonary imaging are diagnostic indicators. Furthermore, a positive tuberculin skin test and TB-IGRA (tuberculosis interferon-γ release assay) can indicate tuberculous infection.

### Other illnesses with chronic cough

In patients with pertussis who have atypical symptoms primarily manifesting as a chronic cough, it is imperative to distinguish their condition from other illnesses that may present similarly. The first step is to rule out pertussis through laboratory testing, which is easier to identify than other diseases are. These illnesses include cough-variant asthma, tracheobronchial foreign bodies, gastroesophageal reflux, and other diagnoses frequently associated with coughing diseases. Cough-variant asthma is often associated with allergies and a prior history of similar coughing episodes. The diagnosis of this condition can be facilitated through allergen testing and pulmonary function tests. Patients with tracheobronchial foreign bodies typically have a history of inhaling a foreign object, accompanied by an irritating cough. Chest imaging may reveal localized atelectasis or emphysema, and recurrent inflammation may affect the same lung lobe or segment. Gastroesophageal reflux is typically characterized by regurgitation of food or acid, and esophagography can be employed for diagnostic purposes. Moreover, even individuals with these diseases are still likely to contract pertussis and may be at a higher risk of infection because of frequent medical visits.

## Treatments

### General therapy

Patients diagnosed with pertussis should be strictly isolated in compliance with respiratory infectious disease protocols to prevent the spread of the disease. Symptomatic supportive treatment is often necessary for managing severe paroxysmal cough and blockage of the respiratory tract by sputum. Nasal feeding may be employed to prevent aspiration and minimize the risk of aspiration pneumonia in infants. In cases with hypoxia, oxygen therapy should be promptly administered to ensure adequate oxygenation. For neonates and small infants, extra caution should be given during nebulization treatments, with close monitoring to ensure timely sputum inhalation post-nebulization and vigilance against the potential risk of asphyxia. Supportive treatment may include providing patients with nutrient-dense and easily digestible foods to maintain their nutritional status.

### Antimicrobial therapy

Empirical antimicrobial therapy is crucial for children of all ages who are clinically or etiologically diagnosed with pertussis and are experiencing the disease's catarrhal stage or paroxysmal spasmodic cough stage. This treatment is essential for eradicating *B. pertussis* strains from the nasopharyngeal region and eliminating the source of infection. Even during the paroxysmal phase, targeted antibiotic therapy can effectively mitigate symptoms in most pediatric patients [21]. Typically, notable improvements are observed within 3–5 days following the completion of treatment [21]. Therefore, we should be alert when initiating antibacterial therapy for patients suspected of having pertussis within 3–4 weeks of onset, particularly those at high risk of developing severe complications. This decision should be made after thoroughly weighing the potential benefits and risks and ensuring adequate communication with the patient and their caregivers.

#### Macrolide antibiotics

Historically, erythromycin, azithromycin, and other macrolide antibiotics have been recommended as the primary antibiotics for treating pertussis [23, 33, 34]. The in vitro susceptibility testing outcomes for *B. pertussis* against various macrolide antibiotics exhibit a high degree of consistency [12, 21, 35, 36], facilitating the selection of macrolides on the basis of drug availability and pediatric patient compliance. Oral administration of azithromycin is generally preferred, with intravenous administration reserved for cases where oral administration is not tolerated [37]. The recommended dosage for azithromycin is 10 mg/kg per dose daily for 5 days, followed by a possible break of 3–4 days before resuming treatment for another 3 days [21, 35, 38]. When erythromycin is prescribed, the recommended dosage for children is 10–15 mg/kg per dose, which does not exceed the maximum daily dose of 2 g, and erythromycin is administered every 8 h for a treatment duration of 14 days. For clarithromycin (oral), the recommended dosage for children aged 1 month or older is 7.5 mg/kg per dose, with a maximum daily dose not exceeding 1 g, twice daily for a treatment duration of 7–14 days.

However, in recent years, clinical strains of *B. pertussis* in China have demonstrated a substantial resistance rate to macrolide antibiotics, as identified by antibiotic susceptibility tests in vitro[12, 21, 35, 36, 39]. Most *B. pertussis* isolates are ERBP strains with high MICs exceeding 256 mg/L [12, 21, 35, 36, 39]. Mi et al. [35] used erythromycin or azithromycin to treat 31 children with ERBP infection and 30 children with erythromycin-sensitive *B. pertussis* (ESBP) infection. They reported that the clearance rates of *B. pertussis* in the nasopharynx after 7 days and 14 days of treatment were 3.2% vs. 33.3% and 22.6% vs. 80.0%, respectively, indicating that ERBP is also resistant to macrolides in vivo and may cause treatment failure in eliminating ERBP strains from the nasopharynx. Consequently, these antibiotics are not recommended as preferred first-line antibiotics for treating pertussis in the Guidelines for Diagnosis and Management and Prevention of pertussis of China (2024 edition) [37]. Here, the experts who drafted this consensus agreed that macrolides should no longer be considered the preferred treatment option. However, if the initial therapeutic approach involves the use of macrolides and a significant deterioration in the patient's condition, if no significant reduction in the bacterial load is observed via PCR or culture during antimicrobial therapy, or if no appreciable improvement is observed after 4–5 days of treatment, it is advisable to switch to alternative antibiotics. Conversely, in cases where infection is confirmed to be ESBP or where empirical treatment with erythromycin, azithromycin, or clarithromycin has yielded positive outcomes, continuation of the same macrolide antibiotic therapy is recommended, typically for a duration of 2 weeks.

Notably, most of the existing data on pertussis resistance to macrolide antibiotics are based on in vitro antibiotic susceptibility tests. Currently, NGS techniques are widely used in clinical settings, and the erythromycin resistance gene may be detected when the *B. pertussis* bacterium is tested. Through the comparison of resistance genes and resistance phenotypes in hundreds of pertussis strains, the expert group behind this consensus has not found any inconsistent strains (data not published), suggesting that the results of erythromycin resistance gene detection are reliable for identifying erythromycin-resistant biological phenotypes.

#### Trimethoprim sulfamethoxazole

Trimethoprim sulfamethoxazole (TMP-SMX) demonstrate robust antibacterial activity against *B. pertussis* both in vivo and in vitro. Considering that ERBP is already widespread in China, alternative antibiotics [34, 37], such as TMP-SMX, are recommended as the preferred oral therapeutic option for children over 2 months of age. The recommended dosage regimen is 24–36 mg/kg per dose, not exceeding a maximum daily dose of 1.92 g, which is administered twice a day for a total duration of 14 days. It is advisable to monitor for potential kidney injury during medication, with an emphasis on maintaining adequate hydration and performing regular urine analysis. Renal function assessment should be conducted if necessary. Notably, TMP-SMX usage is associated with a common incidence of rashes, and treatment should be promptly discontinued in cases of severe anaphylaxis. Additionally, this antibacterial agent is contraindicated in individuals with severe G-6-PD enzyme deficiency, liver or kidney injury, or severe hypersensitivity to sulfamethoxazole.

#### Beta-lactam antibiotics

Beta-lactam antibiotics, including piperacillin, cefoperazone–sulbactam, ceftriaxone, amoxicillin, and ampicillin, exhibit varying degrees of antibacterial activity against *B. pertussis* in vitro [21, 35]. Among these antibiotics, piperacillin and cefoperazone–sulbactam could be cost-effective and readily available options. A study conducted at the Children's Hospital of Zhejiang University School of Medicine evaluated the in vitro drug susceptibility of nearly 1200 strains of *B. pertussis*. The results indicated that piperacillin had an MIC of less than 0.016 mg/L against virtually all strains (data not published). Piperacillin-tazobactam also exhibited remarkable bactericidal activity in eradicating bacteria from the nasopharynx in children [21, 35]. This medication is indicated for intravenous administration in severe cases of pertussis or for use in infants under 2 months of age. The recommended dosage of piperacillin is 40–50 mg/kg per dose, which is administered every 8 h. Piperacillin effectively treats bacterial coinfections, including those caused by *Streptococcus pneumoniae*, *Haemophilus influenzae*, *Moraxella catarrhalis*, or other related pathogens. Typically, the treatment lasts for 1–2 weeks, with the possibility of subsequent TMP-SMX therapy being considered once the patient's condition improves or they reach the age of two months. The clinically proven cefoperazone–sulbactam is also an effective medication for treating pertussis infection [21, 35]. The recommended dosage of cefoperazone–sulbactam is 50–60 mg/kg per dose, which is administered every 12 h. Notably, the efficacy of other beta-lactam drugs has not been thoroughly evaluated. The fact that a beta-lactam antibiotic is not mentioned here does not mean that it is ineffective for pertussis treatment or is as effective as the antibiotics mentioned. Notably, the therapeutic regimens of these beta-lactam antibiotics are clinically effective, primarily on the basis of empirical evidence, and further rigorous clinical trials are necessary to obtain a high-quality, evidence-based evaluation.

*B. pertussis* is a slow-growing bacterium, and antimicrobial treatment requires a relatively long duration, usually about two weeks. Any premature discontinuation of medication may result in a relapse of pertussis symptoms. Therefore, unless the etiological test is negative after treatment, it is not recommended to interrupt antimicrobial treatment, even after 5–7 days, even if there are significant clinical improvements. Other antibiotics can replace antibiotics during treatment because of side effects or other reasons. It is advisable to conduct DNA testing or bacterial culture assessments at intervals of 5–7 days throughout the therapeutic process. Evidence of clinical symptom improvement, along with a reduction in the quantity of bacterial colonies as determined by culture or a decrease in nucleic acid copies measured by qPCR, indicates the effectiveness of the treatment. The duration of medication may be shortened once the results of the etiology test return negative.

#### Antimicrobial therapy for close contacts

In the management of newborn and infant patients, antimicrobial therapy should be duly considered for their close contacts. As a primary treatment modality, it is advisable to prescribe TMP-SMX orally to etiologically positive family members or caregivers. Fluoroquinolones may serve as a viable alternative for adults with high-TMP-SMX allergies. Infants who have had close contact with a person with pertussis and cannot wholly interrupt this contact should be promptly administered sensitive antibacterial agents for prophylactic purposes, adhering to the medication mentioned above and the treatment protocol. The targeted population includes (1) infants aged 3 months or younger, (2) children attending childcare facilities who have not received four doses of the pertussis vaccine, and (3) household members or healthcare workers responsible for providing care to infants younger than 3 months.

#### Antimicrobial therapy for “asymptomatic infections”

In individuals who have no obvious symptoms but are positive for pertussis, close observation, regular follow-up, and periodic retesting are advisable [40]. Furthermore, patients exhibiting recurrently positive etiological tests, especially those with positive bacterial culture results, indicating active infections, should receive antimicrobial therapy, particularly if they are in constant interaction with individuals who are at high risk of developing severe pertussis.

### Intensive treatment for severe pertussis

Severe pertussis patients necessitate intensive treatment alongside antibiotic therapy. Nevertheless, a standardized approach to selecting and timing intensive treatment for pertussis remains elusive, and its impact on prognosis remains inconclusive across various studies. Consequently, the modalities of intensive treatment are currently in the exploratory phase.

1. Mechanical ventilation therapy is necessary when apnea and respiratory failure persist despite the administration of standard treatment measures.
2. Treatment of hyperleukemia is essential, as it poses a significant risk for severe pertussis and even death [24, 25, 27, 29]. Effectively reducing the total count of peripheral blood leukocytes can significantly improve the prognosis of patients. Currently, two primary treatment methods are leukapheresis and therapeutic plasma exchange. Among these, therapeutic plasma exchange is a more established, safer, and easier-to-perform approach than leukapheresis. In particular, therapeutic plasma exchange is highly recommended in the following scenarios, especially for infants younger than 60 days who are suffering from pertussis[24, 41]: (a) Whose WBC count exceeds 25 × 10^9^/L and the lymphocyte count surpasses 12 × 10^9^/L, complicated by conditions such as heart failure, pulmonary hypertension, or organ failure; (b) in cases where the WBC count increases above 48 × 10^9^/L, the lymphocyte count exceeds 15 × 10^9^/L; (c) whose the WBC count exceeds 30 × 10^9^/L, it increases by more than 50% within a 24-h period, and the lymphocyte count also exceeds 15 × 10^9^/L. By adhering to these recommendations, therapeutic plasma exchange can be effectively utilized to manage hyperleukemia and mitigate its associated risks.
3. The management of pulmonary hypertension involves targeted pulmonary vasodilator therapies, including endothelin receptor antagonists, prostacyclin analogs, and phosphodiesterase type 5 inhibitors, which result in hemodynamic and functional improvements in children [42]. Inhaled NO (iNO) is commonly used in the acute management of pulmonary arterial hypertension, which presents with hemodynamic instability and right heart failure. The use of iNO in term and near-term infants for treating persistent pulmonary hypertension (PPHN) in newborns has been well studied and documented through several double-blinded placebo-controlled trials [43, 44]. Typically, the initial dose begins at 20 parts per million (ppm), which is promptly reduced to 5 ppm once the oxygenation status has improved. Following this, gradual tapering to 1 ppm precedes discontinuation [45]. It is generally advisable to limit the duration of treatment to no more than 5 days. However, the therapeutic effect of iNO on pertussis-induced pulmonary hypertension is still uncertain [24].
4. The optimal timing for initiating extracorporeal membrane oxygenation (ECMO) support in patients with severe pertussis remains a topic of ongoing debate, and the overall survival rate associated with this therapy remains low. It is advisable to initiate ECMO earlier, rather than as a late remedial measure, in pertussis patients with multiple risk factors that predict poor ECMO outcomes. These risk factors include young age, low ratios of partial pressure of oxygen to fraction of inspiration oxygen (PaO_2_/FiO_2_), the need for vasoactive medications, the presence of a neuronal system or infectious complications, pulmonary hypertension, and rapid disease progression (requiring intubation and ECMO support within 24 h of hospital admission) [46]. In particular, patients with severe pulmonary hypertension, where hemodynamic collapse may occur rapidly, are strong candidates for earlier ECMO initiation [47, 48].
5. Intravenous administration of pertussis immunoglobulin (P-IVIG) at a dosage of 15 mL/kg daily for 1–2 days.

### Chinese medicine treatment

Traditional Chinese Medicine for treating pertussis principally focuses on purging the lungs, clearing heat, resolving phlegm, and reducing adverse flow. Clinically, pertussis was divided into the initial cough stage, paroxysmal cough stage, and recovery stage, advocating for lung-dispelling, lung-purging, and lung-nourishing treatments, respectively.

During the initial cough stage, the treatment principal is to disperse wind and promote lung ventilation, with the formula of choice being the modified Mulberry Leaf and Chrysanthemum Drink. Commonly used medicinal herbs include mulberry leaf, chrysanthemum flower, platycodon root, stemona root, bitter apricot seed, perilla leaf, loquat leaf, forsythia fruit, cowherb seed, mint, and licorice.

In the paroxysmal cough stage, the treatment principal is to purge the lungs and resolve phlegm, with the formula of choice being the combined Mulberry Bark Decoction and Tussilago and Jujube Lung-Draining Decoction. Commonly used medicinal herbs include mulberry bark, scutellaria root, fritillaria bulb, pinellia rhizome, stemona root, perilla seed, bitter apricot seed, scutellaria root, coptis rhizome, coicis seed, gardenia fruit, and jujube.

During the recovery stage, the treatment principal is to nourish yin and boost qi, with the formula of choice being the combined Ginseng and Schisandra Decoction and Ophiopogon and Oatstraw Decoction. Commonly used medicinal herbs include ophiopogon, codonopsis root, oatstraw, polygonatum root, poria, atractylodes macrocephala, mulberry leaf, and trichosanthes root.

Traditional Chinese Medicine emphasizes individualized treatment. Therefore, in this consensus guideline, we recommend herbal medicines without specifying dosages.

### Criteria for the removal of quarantine

Respiratory isolation may be terminated when any of the following criteria are fulfilled: (1) The patient has completed a course of sensitive antibiotics for at least 5 days [34, 49], leading to a notable improvement in their clinical manifestations. (2) The patient has been experiencing paroxysmal cough for a duration exceeding 21 days [34]. (3) No positive results are obtained from nasopharyngeal swab cultures, nasopharyngeal or oropharyngeal swab testing for *B. pertussis* DNA. Children who meet criteria (1) and (2) for removal of quarantine but whose nasopharyngeal swab culture results are positive are advised to continue antimicrobial therapy. Additionally, it is strongly recommended that they wear masks upon resuming school attendance.

## Prevention

### General prevention

Pertussis, is a respiratory infection caused by the bacterium *B. pertussis*, exhibiting a restricted in vitro survival capacity. The implementation of strategies, such as social distancing, emphasizing proper hand hygiene, and wearing masks, can effectively minimize the risk of contracting infection when patients are in contact with pertussis patients.

### Enhanced post-exposure drug prophylaxis

Post-exposure drug prophylaxis is recommended for high-risk individuals who may develop severe pertussis following infection (see the antimicrobial therapy for close contacts section). However, it is not advisable to administer antibiotic prophylaxis in close contact with pertussis patients in the general population.

### Vaccination

Currently, the pertussis vaccines used in China are combination vaccines composed of diphtheria toxoid, tetanus toxoid, and acellular pertussis (DTaP) vaccines or DTaP vaccines combined with Haemophilus type b conjugate vaccines and/or inactivated poliomyelitis vaccines. According to the national immunization schedule, children are required to receive four doses of the DTaP vaccine at 3, 4, 5, and 18 months of age. A meta-regression modeling study using discrete time points since vaccination to estimate the acellular pertussis vaccines effectiveness of initial childhood series is 91% (95% CI: 87% to 95%) and declines at 9.6% annually [50]. Therefore, booster vaccination with the pertussis vaccine is suggested to strengthen immunity for all vulnerable individuals in areas where pertussis is epidemic [51].

In summary, the resurgence of pertussis in China has recently emerged as a critical concern in pediatric infectious diseases [51]. According to the available data, the majority of confirmed cases involve children aged 5–9 years, whereas severe manifestations are still predominantly observed in neonates and young infants. Clinicians must promptly identify severe cases and implement interventions to mitigate the disease risk of pertussis. This consensus states the diagnostic criteria for pertussis, high-risk factors, and severe pertussis. Macrolides are recommended for treating ESBP infections, whereas TMP-SMX oral therapy is the initial treatment option for children older than 2 months. For infants younger than 2 months, severely ill patients, or those exhibiting a high degree of sulfonamide allergy, intravenous administration of piperacillin or cefoperazone–sulbactam is advised. Expert consensus provides comprehensive guidance and a reference for the diagnosis and treatment of pertussis in children. Notably, while some recommendations in this consensus are grounded in extensive clinical experience, further high-quality, evidence-based research through prospective randomized clinical trials is necessary to assess the specific efficacy of TMP-SMX and piperacillin, ultimately ensuring the scientific rigor and therapeutic effectiveness of the proposed treatment strategies. The diagnosis and treatment flow diagram for children with whooping cough is summarized in Fig. 1.

There are a few points that remain unresolved and have yet to achieve consensus and needs further researches to provide evidences:

1. Does asymptomatic *B. pertussis* infection exist? There are individuals who, despite actively undergoing monitoring and testing positive for *B. pertussis* nucleic acid or culture in their respiratory secretions, exhibit no apparent symptoms. Some experts hypothesize that these individuals are infected with *B. pertussis*, albeit with symptoms so mild that they are often misinterpreted as asymptomatic. Further research, through retrospective analysis, could determine whether these patients were indeed infected with *B. pertussis* by monitoring their pertussis antibody levels and assessing whether an immune response was triggered.
2. Should it be labeled as bacteremia or nucleic acidemia? Clinically, there have been instances where NGS has detected *B. pertussis* nucleic acid in the bloodstream of patients with severe pertussis. The consensus among most experts is that this finding merely signifies a state of nucleic acidemia, rather than bacteremia, and thus cannot serve as a robust predictor of high risk for severe pertussis.
3. This consensus does not distinguish between severe pertussis and critically ill pertussis patients. Future endeavors must be directed toward further examining these lingering issues.

## Data Availability

Not applicable for this paper.
